# Draft Genome Sequences of Two Novel Cellulolytic Streptomyces Strains Isolated from South African Rhizosphere Soil

**DOI:** 10.1128/genomeA.00632-18

**Published:** 2018-06-28

**Authors:** Mobolaji F. Adegboye, Briallen Lobb, Olubukola O. Babalola, Andrew C. Doxey, Kesen Ma

**Affiliations:** a Department of Biology, University of Waterloo, Waterloo, Ontario, Canada; b Food Security and Safety Niche Area, Faculty of Natural and Agricultural Sciences, North-West University, North West, South Africa

## Abstract

We report here the draft genome sequences of two novel strains of Streptomyces (NWU339 and NWU49) isolated from South African rhizosphere soils. Both strains were found to possess strong cellulolytic activity and contain numerous putative cellulase genes. Both genomes possess benzoate degradation pathways, while NWU49 contains the genomic potential for enediyne biosynthesis.

## Abstract (precis)

We report here the draft genome sequences of two novel strains of Streptomyces (NWU339 and NWU49) isolated from South African rhizosphere soils. Both strains were found to possess strong cellulolytic activity and contain numerous putative cellulase genes.

## GENOME ANNOUNCEMENT

Streptomyces species are known for their diverse metabolic potential, wide range of antibiotic biosynthesis capabilities, and ability to degrade unique compounds, such as lignocellulose, keratin, xylan, pectin, cellulose, lignin, chitin, and styrene (1–4). They also produce various hydrolytic enzymes, such as amylase, lipase, esterase, gelatinase, xylanase, and cellulases. Cellulases can be used for the hydrolysis of lignocellulose to fermentable sugars which can be used as feedstock for the production of biofuels that have been proven to be environmentally friendly, help reduce dependence on fossil fuel, and serve as an alternative for declining petroleum reservoirs (5). The isolation of environmental Streptomyces species capable of lignocellulose degradation is therefore of considerable interest.

Two novel Streptomyces strains (NWU339 and NWU49) were isolated from the rhizosphere of maize in North West Province, South Africa. Both strains were capable of growing on polymeric carbohydrate substrates, such as starch, xylan, and cellulose. Streptomyces sp. strains NWU339 and NWU49 were cultured using starch casein agar (6). Genomic DNA (50 ng) was extracted using the Wizard genomic DNA purification kit from the Promega Corporation. Sequencing libraries were prepared using the Nextera DNA sample preparation kit (Illumina). Sequencing was performed on an Illumina HiSeq platform, and genome assembly was performed using NGen version 14 with Q25 trimming. The assembly of NWU339 produced 169 contigs, resulting in a draft genome of 9,425,309 bp, with a GC content of 70.8%. The assembly of NWU49 produced 97 contigs, resulting in a draft genome of 8,905,076 bp, with a GC content of 72.3%. Genome annotation was then performed using Prokka version 1.12 (databases downloaded 26 January 2018).

The genome of NWU339 encodes 8,776 protein-coding sequences (CDSs), 8 rRNA genes, and 88 tRNAs. Phylogenetic analysis of taxonomic marker genes using MetAnnotate (7) revealed NWU339 to be a novel Streptomyces strain with 97% 16S rRNA identity to Streptomyces poonensis NRRL B-2319. The genome of NWU49 encodes 8,021 CDSs, 7 rRNA genes, and 100 tRNAs. NWU49 possesses 98% 16S rRNA identity to Streptomyces ghanaensis NBRC15414. A comparison of NWU49 to the genome of Streptomyces ghanaensis ATCC 14672 using the Genome-to-Genome Distance Calculator 2.1 (8) indicates that they are likely the same species (96.27% probability that DNA-DNA hybridization is greater than 70% using formula 2).

NWU339 contains 15 putative cellulase-related genes, including 5 predicted subtypes of endoglucanases, 3 subtypes of exoglucanases, and 4 subtypes of beta-glucosidases. KEGG analysis suggests the genomic potential for metabolic pathways to degrade benzoate, toluene, and xylene. NWU49 contains 18 putative cellulase-related genes, including 8 predicted subtypes of endoglucanases, 3 subtypes of exoglucanases, and 4 subtypes of beta-glucosidases. NWU49 contains pathways for the degradation of sphingosine and *trans*-cinnamate and for the biosynthesis of polyamine and trehalose. Complete predicted pathways in NWU49 include the degradation of benzoate and the biosynthesis of the core molecule for enediyne, an anticancer metabolite.

### Accession number(s).

The whole-genome shotgun projects for Streptomyces sp. NWU339 and Streptomyces sp. NWU49 have been deposited in GenBank under the accession numbers QFRK00000000 and QFXB00000000, respectively. The versions described in this paper are QFRK01000000 and QFXB01000000, respectively. The raw reads were deposited in the Sequence Read Archive (SRA) under the accession number SRP148117.
